# Using Machine Learning Techniques to Develop Risk Prediction Models for the Risk of Incident Diabetic Retinopathy Among Patients With Type 2 Diabetes Mellitus: A Cohort Study

**DOI:** 10.3389/fendo.2022.876559

**Published:** 2022-05-17

**Authors:** Yuedong Zhao, Xinyu Li, Shen Li, Mengxing Dong, Han Yu, Mengxian Zhang, Weidao Chen, Peihua Li, Qing Yu, Xuhan Liu, Zhengnan Gao

**Affiliations:** ^1^ Department of Endocrinology, Dalian Municipal Central Hospital, Dalian, China; ^2^ Infervision Institute of Research, Beijing, China; ^3^ Graduate School of Art and Science, Yale University, New Haven, CT, United States

**Keywords:** type 2 diabetes mellitus, diabetic retinopathy, machine learning, XGBoost model, cohort study

## Abstract

**Objective:**

To construct and validate prediction models for the risk of diabetic retinopathy (DR) in patients with type 2 diabetes mellitus.

**Methods:**

Patients with type 2 diabetes mellitus hospitalized over the period between January 2010 and September 2018 were retrospectively collected. Eighteen baseline demographic and clinical characteristics were used as predictors to train five machine-learning models. The model that showed favorable predictive efficacy was evaluated at annual follow-ups. Multi-point data of the patients in the test set were utilized to further evaluate the model’s performance. We also assessed the relative prognostic importance of the selected risk factors for DR outcomes.

**Results:**

Of 7943 collected patients, 1692 (21.30%) developed DR during follow-up. Among the five models, the XGBoost model achieved the highest predictive performance with an AUC, accuracy, sensitivity, and specificity of 0.803, 88.9%, 74.0%, and 81.1%, respectively. The XGBoost model’s AUCs in the different follow-up periods were 0.834 to 0.966. In addition to the classical risk factors of DR, serum uric acid (SUA), low-density lipoprotein cholesterol (LDL-C), total cholesterol (TC), estimated glomerular filtration rate (eGFR), and triglyceride (TG) were also identified to be important and strong predictors for the disease. Compared with the clinical diagnosis method of DR, the XGBoost model achieved an average of 2.895 years prior to the first diagnosis.

**Conclusion:**

The proposed model achieved high performance in predicting the risk of DR among patients with type 2 diabetes mellitus at each time point. This study established the potential of the XGBoost model to facilitate clinicians in identifying high-risk patients and making type 2 diabetes management-related decisions.

## Introduction

DR is the most common microvascular complication of diabetes mellitus. It has been demonstrated to be a leading cause of preventable blindness in the working-age population in most countries (1).

The American Academy of Ophthalmology (AAO), in 2019, stated that the prevalence of DR among diabetic patients worldwide is about 34.6%. 10.2% (28 million) diabetic patients suffer from vision-threatening DR (2). Effective management of DR requires a deep understanding of the predisposing factors, early diagnosis, and timely therapeutic intervention. Early identification of patients at risk of developing DR is the key to effective intervention, which is significant in reducing the progression of DR and thereby reducing the risk of blindness (3). Moreover, individual patients can be stratified according to different risk levels and get optimal treatment. Due to no typical symptoms in the early stage of the disease, however, most patients with DR may not seek medical evaluation until progression to the proliferative stage, resulting in irreversible visual damage (4). Therefore, methods for accurate prediction of the risk of DR are in urgent need.

At present, several DR risk prediction models based on cross-sectional studies have been developed (5–9). Deep learning algorithms were also applied (10). Although these models can predict the occurrence of DR at the index date, they cannot predict DR occurrence and development of the same patient at designated time points in the future. This will obviously restrict their clinical application. Similarly, based on the clinical characteristics related to the occurrence or development of DR, several models have been developed for optimization of the screening interval in DR screening (11, 12). However, due to the small number of cases in the studies, the proposed models have not been fully validated so far.

On the other hand, several studies investigated the pathogenesis and risk factors of DR to provide guidance for DR management. Epidemiological studies have shown that age, course of diabetes, hemoglobin A1c(HbA1c), fasting blood glucose (FBG), blood pressure, blood lipids, body mass index (BMI), smoking, proteinuria, and several others are all risk factors for DR (13, 14). Among them, duration of diabetes and hyperglycemia were demonstrated as strong risk factors for the occurrence and development of DR (15, 16). However, patients without DR were hardly unusual among those suffering from diabetes for a long time (17). The influences of other factors in DR occurrence also need to be proven. Further studies are required to elucidate the correlation and thus construct standard procedures for the management of this disease.

In this retrospective cohort study, we collected electronic health record data from hospitalized patients with type 2 diabetes and established the prediction model for future risks of DR based on a machine learning (ML) algorithm. To our knowledge, this is the first study to predict the occurrence of DR at each follow-up time point in up to 10 years. We also explore the risk factors that may affect the occurrence of DR and hope this work can provide a basis for further studies concerning the prevention and management of DR.

## Materials And Methods

### Study Subjects

The study protocol was approved by the Ethics Committee of Dalian Medical University Affiliated with the Central Hospital of Dalian. The ethics committee waived the requirement of written informed consent for all patients. The data did not contain any direct patient identifiers. The study adhered to the tenets of the Declaration of Helsinki.

### Datasets

In this retrospective cohort study, we recruited inpatients admitted to the Department of endocrinology of Dalian Medical University Affiliated with the Central Hospital of Dalian from January 2010 to September 2018. Patients who met the following criteria were included in the current study: 1) Age≥18 years with the diagnosis of type 2 diabetes; 2) be hospitalized at least once over the follow-up period; 3) no presence of DR at the time of the first hospitalization. Patients with other types of diabetes (type 1 diabetes, special type diabetes, or gestational diabetes) or previous eye diseases (cataract, glaucoma, or other eye diseases) were excluded. With the criteria mentioned above, 7943 patients were selected for this study, including 1692 patients diagnosed with DR in the follow-up period (DR group) and 6521 control ones without DR (non-DR group). These patients were randomly divided into a training set (n=5559) and a test set (n=2384).

### Diagnostic Criteria

Diagnostic criteria of type 2 DM are according to 1999 WHO diagnostic criteria (18). DR was examined by dilated fundus examination by ophthalmologists and endocrinologists together. Diagnostic criteria of DR case meet the Diabetic Retinopathy preferred practice pattern (PPP)-2019 guideline (19). The grading of DR in this study was based on the International Clinical Diabetic Retinopathy Severity Scales (20): Grace-1 for no apparent retinopathy; Grace-2 for mild non-proliferative diabetic retinopathy (NPDR), which includes microaneurysms only; Grace-3 for moderate NPDR, which includes more than just microaneurysms but less than severe nonproliferative DR; Grace-4 for severe NPDR (any of the following can be diagnosed as Grace-4: more than 20 intraretinal hemorrhages in each of 4 quadrants, definite venous beading in 2+ quadrants, Prominent intraretinal microvascular abnormalities in 1+ quadrant); and Grace-5 for proliferative diabetic retinopathy (PDR). The individual diagnoses were described according to the diagnosis and staging of the worse one between the two eyes.

### Variable Selection

Patients’ information on demographics, medical history, medication, and laboratory data was extracted from the EHR at baseline and used as candidate predictor variables for developing DR (**Tables S2, S3**). These variables are commonly associated with the risk of DR, including 11 categorical variables and 20 continuous variables. The smoking status was defined as: 1) Current smokers: Patients who smoked more than 100 cigarettes and had not quit smoking at the index date; 2) Ex-smokers: Patients who smoked more than 100 cigarettes and stopped smoking 30 days before index date; 3) Never smokers: Patients who had never smoked. The drinking status was defined as: 1) Current drinkers: Patients who have been drinking or have not abstained from alcohol for more than one year; 2) Ex-drinkers: Patients who did not drink or had abstained from drinking for more than one year; 3) Never-drinks: Patients who had never drunk. Hypertension was defined as a systolic blood pressure of ≥140 mmHg and/or a diastolic blood pressure ≥90 mmHg, measured twice or more on different days, whether antihypertensive drugs were used or not (21). Blood samples were collected the next morning after hospital admission with at least 12-h fasting. HbA1c was measured using the glycosylated hemoglobin analyzer (TOSOH company of Japan, HLC-723G8). Four items of blood lipids (high-density lipoprotein cholesterol (HDL-C), TC, LDL-C, and TG), alanine transaminase (ALT), aspartate transaminase (AST), gamma-glutamyl transpeptidase(γ-GT), serum creatinine (Scr), SUA, and FBG were detected by automatic biochemical analyzer (Siemens, Germany, ADVIA2400 biochemical system). The eGFR was calculated according to the CKD-EPI formula (22). BMI was calculated as weight (kg) divided by the square of height in meters (m^2^); Blood pressure was measured on the right arm in sitting position three times consecutively at 5-min intervals, with the mean of the three measurements used for analysis.

### Machine Learning Models Construction

Machine learning-based algorithms were selected according to the criteria below. First, the algorithms should show evaluation indicators based on mixed data type, including numerical variables and categorical variables. Second, the algorithms should have a wide range of applications and a history of successful usage in related fields. Based on the above criteria, we selected five machine learning algorithms, Random Forest (RF) (23), Extreme Gradient Boosting (XGBoost) (24), Logistic Regression (LR) (25), Support Vector Machine (SVM) (26), and K-Nearest Neighbor (K-NN) (27), to build the models with all the variables as predictors in this study. We used the GridSearchCV method to select the optimal key hyper-parameters of the five models as shown in **Table S3**, and other hyper-parameters were set as default. The models were then tested and internally validated by fivefold cross-validation.

### Statistical Analysis

All statistical analyses were performed by using R statistical and computing software version 4.0.2 (http://www.r-project.org/). *Chi*-square test was used for categorical variables, and two-sample *t*-test for continuous variables to compare the variables between DR group and non-DR group. Two-tailed hypothesis testing was used. *P*<0.05 indicated a statistically significant result.

Performance of the proposed models was assessed mainly in terms of sensitivity, specificity, positive predictive value (PPV), negative predictive value (NPV), and accuracy. We assessed these metrics at the optimized prediction threshold identified by the Youden index. To compare performance across the models, we also calculated the area under the receiver operating characteristic curve (*AUC*). Significance testing and *LASSO* penalty were employed to identify clinical features that are associated with a high risk of DR. Nomograms were built based on the results of multivariate logistic regression analysis. The performance of the nomograms was measured using Harrell’s concordance index (*C*-index).

## Results

### Study Population

Baseline characteristics of 7943 patients with type 2 diabetes, including 1692 patients diagnosed as DR (DR group) and 6521 without DR signs (non-DR group) during the follow-up period, are presented in **Table S4**. The mean age of the patients was 63.54 years, and 45.7% were women. Mean follow-up time was 3.139 ± 2.243 years, and mean duration of diabetes mellitus was 1.449 ± 2.756 years. To further validate the performance of the model, 829 multipoint data from different follow-up time points were collected from 508 patients in the test set (supplementary test set).

### Prediction Performances of the ML Models

**Figure 1:**
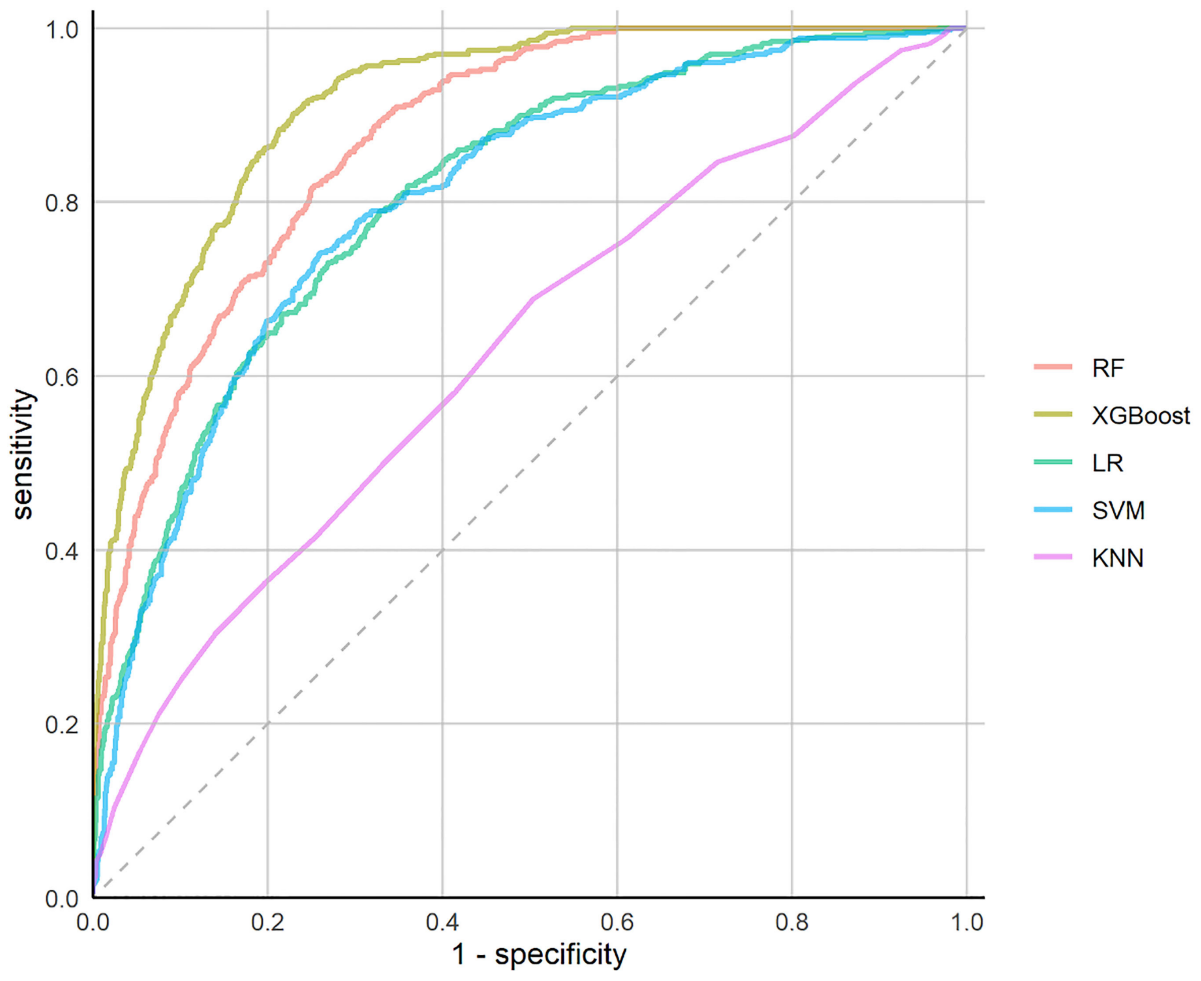
Receiver Operating Characteristics (*ROC*) curves of the ML models.

The performances of the prediction models were assessed in terms of predefined evaluation metrics and *ROC* (**Table 1** and **Figure 1**). The XGBoost model outperformed the other models with the highest *AUC* (0.913; 95% confidence interval (*CI*), 0.901-0.925), highest accuracy (79.9%; *CI*, 76.9%-83.5%), highest sensitivity (90.2%; *CI*, 84.8%-94.9%), highest specificity (77.1&; *CI*, 72.1%- 82.7%), highest PPV (51.6%; *CI*, 47.9%-57.5%) and highest NPV (96.7%; *CI*, 95.2%-98.1%).

**Table 1:** The performance metrics of the cross-validated machine learning algorithms on the test data.

Model	AUC (95% CI)	Accuracy (95% CI)	Sensitivity (95% CI)	Specificity (95% CI)	PPV (95% CI)	NPV (95% CI)
RF	0.872 (0.857, 0.887)	0.764 (0.703, 0.785)	0.817 (0.793, 0.925)	0.749 (0.647, 0. 776)	0.469 (0.41, 0.498)	0.938 (0.931, 0.971)
XGBoost	0.913 (0.901, 0.925)	0.799 (0.769, 0.835)	0.902 (0.848, 0.949)	0.771 (0.721, 0. 827)	0.516 (0.479, 0.575)	0.967 (0.952, 0.981)
LR	0.808 (0.787, 0.828)	0.731 (0.659, 0.789)	0.73 (0.636, 0.852)	0.731 (0.61, 0. 828)	0.424 (0.368, 0.504)	0.909 (0.893, 0.94)
SVM	0.802 (0.781, 0.823)	0.742 (0.702, 0.776)	0.74 (0.677, 0.805)	0.742 (0.681, 0.803)	0.437 (0.398, 0.482)	0.913 (0.901, 0.93)
K-NN	0.629 (0.601, 0.656)	0.537 (0.522, 0.752)	0.689 (0.303, 0.73)	0.496 (0.478, 0.868)	0.27 (0.261, 0.399)	0.855 (0.82, 0.872)

### Prediction Performance of the XGBoost Model in Different Follow-Up Periods

The XGBoost model was further assessed as a potential tool to predict the occurrence of DR (**Table 2**). According to follow-up intervals, the test set was divided into eight categories, respectively, each year of 1 to 8 years and more than 8 years. The highest *AUC* of 0.966 (95% *CI*, 0.955-0.977) and highest accuracy of 87.7% (95% *CI*, 85.5%-93.3%) were achieved in patients who were followed up within the timeframe of 1 to 2 years.

**Table 2:** The performance metrics of the XGBoost model for different follow-up times.

*Follow-up period	Patient number	DR patient number	AUC(95% CI)	Accuracy (95% CI)	Sensitivity (95% CI)	Specificity(95% CI)	PPV(95% CI)	NPV(95% CI)
(1, 2)	1025	93	0.966 (0.955, 0.977)	0.877 (0.855, 0.933)	0.978 (0.925, 1)	0.867 (0.841, 0.931)	0.423 (0.382, 0.578)	0.998 (0.992, 1)
(2, 3)	352	92	0.834 (0.788, 0.879)	0.733 (0.668, 0.801)	0.848 (0.739, 0.957)	0.692 (0.577, 0.812)	0.494 (0.435, 0.592)	0.928 (0.894, 0.977)
(3, 4)	283	78	0.867 (0.817, 0.918)	0.77 (0.728, 0.873)	0.859 (0.654, 0.949)	0.737 (0.659, 0.932)	0.554 (0.503, 0.797)	0.932 (0.873, 0.972)
(4, 5)	242	70	0.86 (0.809, 0.911)	0.785 (0.707, 0.851)	0.8 (0.686, 0.957)	0.779 (0.628, 0.901)	0.596 (0.496, 0.742)	0.905 (0.872, 0.977)
(5, 6)	153	49	0.835 (0.765, 0.905)	0.765 (0.719, 0.869)	0.776 (0.531, 0.898)	0.76 (0.654, 0.962)	0.603 (0.538, 0.879)	0.878 (0.809, 0.943)
(6, 7)	129	38	0.876 (0.814, 0.938)	0.837 (0.705, 0.899)	0.711 (0.605, 0.974)	0.89 (0.604, 0.967)	0.73 (0.5, 0.897)	0.88 (0.85, 0.983)
(7, 8)	116	45	0.917 (0.867, 0.966)	0.845 (0.759, 0.897)	0.822 (0.733, 0.933)	0.859 (0.732, 0.901)	0.787 (0.655, 0.841)	0.884 (0.825, 0.955)
(8, 11)	84	43	0.858 (0.779, 0.936)	0.798 (0.738, 0.881)	0.791 (0.698, 1)	0.805 (0.561, 0.927)	0.81 (0.691, 0.92)	0.786 (0.72, 1)

*Follow-up period indicated the corresponding time frame that included the former time point but not the latter one.

### Information Contributions of the Features

**Figure 2:**
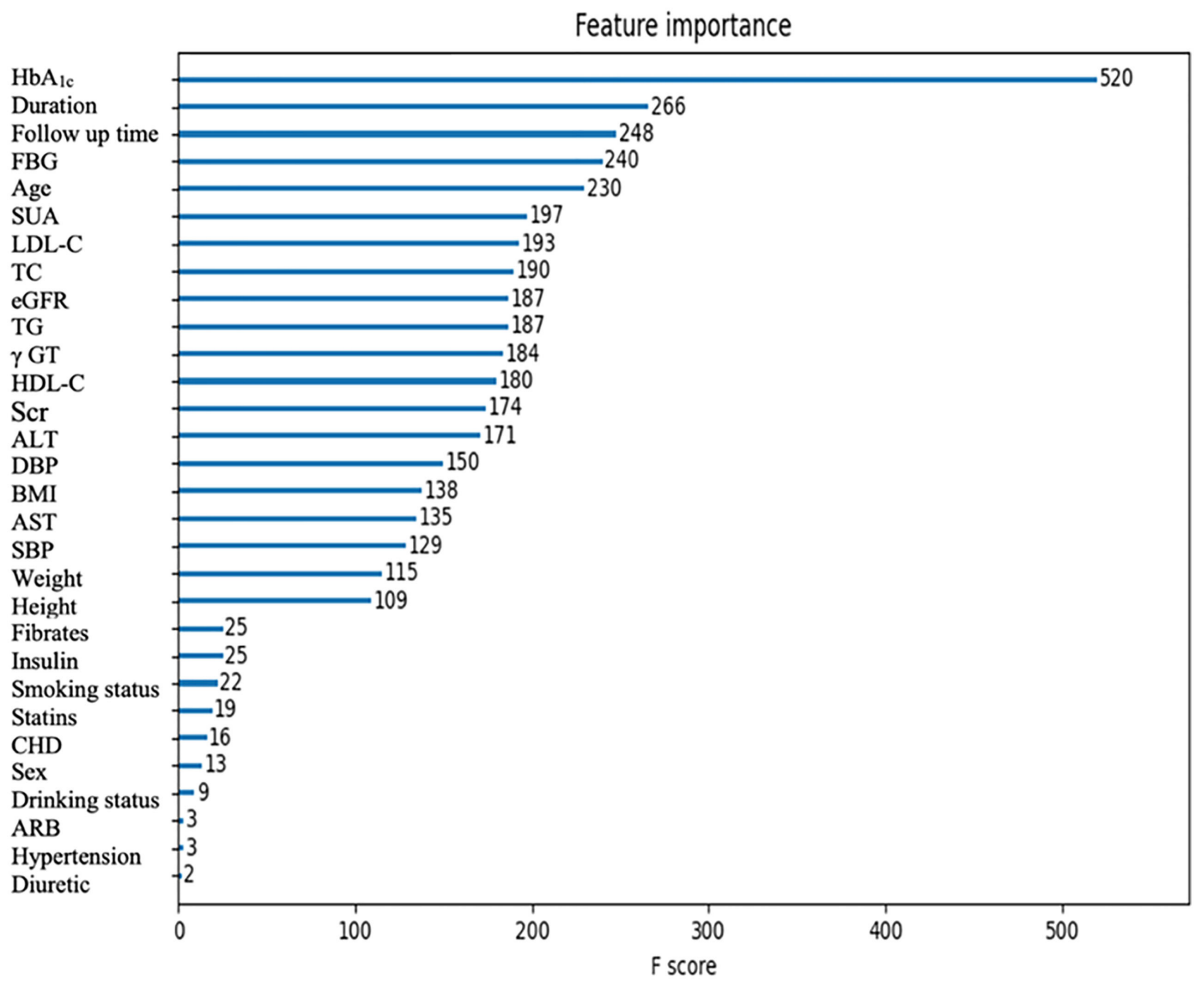
The importance scores of the features calculated by XGBoost.

The importance scores calculated by XGBoost are shown in **Figure 2**. Among all the features, the top five ones (HbA1c, duration, follow-up time, FBG, and age) contributed the most to identifying cases with DR risk in type 2 diabetes patients. In addition, the importance scores calculated by RF, which is the next-best performing model, are shown in **Figure S1**.

### Subgroups Analysis Based on Follow-Up Period and DR Severity Level

The XGBoost model was further evaluated on the supplementary test set. Multipoint data of the same patient were used as new cases. The model showed an improved performance with an *AUC*, accuracy, sensitivity, specificity, *PPV*, and *NPV* of 0.922 (95%*CI*, 0.912-0.932), 81.5% (95% *CI*, 80.4%-84.2%), 92.6% (95% *CI*, 87.1%-94.2%), 76.6% (95% *CI*, 74.9%-82.4%), 63.6% (95% *CI*, 62.1%-68.8%), and 95.9% (95% *CI*, 93.5%-96.8%) respectively. Detailed attributes are presented in **Table S5**. Age, diuretic, fibrates, eGFR, HbA1c, duration, and follow-up time were significantly different between the cases with correct predictions and those with incorrect predictions. We then measured the time to diagnosis. The XGBoost model achieved an average of 2.895 ± 2.104 years prior to the first diagnosis by the clinical diagnosis method.

Subgroups analyses based on follow-up period and DR severity level were respectively conducted to assess the potential application of the model in clinical scenarios (**Supplementary Figure S4**). With the increase in follow-up time, the true positive prediction rate of the XGBoost model increases (**Supplementary Figure S4A**). The subgroup of patients followed up within the time frame of 7 to 8 years exhibited the highest positive prediction rate (100.0%). Data of all the others were higher than 87%. As for different DR severity subgroups, the model performed better for grade 3, reaching the highest positive prediction rate of 97.56% (**Supplementary Figure S4B**).

### Nomograms for Predicting DR Risk of Patients With Type 2 Diabetes

Nomograms were developed to depict the association between the clinical variables and the occurrence of DR. After feature selection, we obtained 16 features including γGT, AST, SBP, eGFR, age, HbA1c, FBG, duration, follow-up time, insulin, diuretic, statins, fibrates, hypertension, smoking status, and drinking status. A classic LR model and an LR model integrated with the machine learning output were constructed (**Supplementary Figure S2, S3**). The C-index of the integrated LR model was 0.921, whereas that of the classic LR model was 0.836.

## Discussion

In this study, we constructed five ML-based models for the risk of developing DR among patients with type 2 diabetes using EHR data, the XGboost model of which demonstrated the best performance. We further evaluated the predictive value of this model and found that it can accurately predict the occurrence of DR at each time point over 10 years. According to feature importance analysis, five risk factors, SUA, LDL-C, TC, eGFR, and TG, were recognized as important indicators of DR for the first time. Moreover, analysis of data from multiple time points revealed that predicting DR risk with the XGBoost model would obviously reduce the amount of time needed for the diagnosis of DR.

XGBoost algorithm is known for its good scalability and high running speed, which is thus commonly used and developed in recent years (28–30). Specifically, the XGBoost model provides the following advantages. First, the XGBoost algorithm reduces the order of magnitude of features while retaining all the features by introducing regularization terms into the objective function to avoid over-fitting. Secondly, the algorithm also used the random forest algorithm for reference, which can not only reduce the over-fitting problem but also reduce the computational complexity. We hypothesized that these reasons would be responsible for the XGBoost algorithm’s outstanding prediction ability for DR risk compared with the other four methods adopted in this study.

There were several studies gaining insights into DR risk prediction, some of which developed ML-based models. However, most of them were cross-sectional studies. In a study in Iran (31), 3734 diabetic patients were included to build a logistic regression model with an *AUC* of 0.704. The input variables included sex, age, diabetes duration, BMI, blood pressure (BP), HbA1c, FPS, cholesterol, and triglycerides. In the cross-sectional study by Ein hoh et al. (7) in South Korea, a *LASSO* prediction model was constructed for predicting the risk of DR, reaching an *AUC* of 0.82 and an accuracy of 75.2%. 490 diabetic patients were selected for the study, and several variables (age, sex, smoking history, drinking history, waist circumference, BMI, physical exercise status, medication history, blood pressure, and relevant laboratory results) were used as input. In another cross-sectional study in Taiwan Province of China (6), 536 patients with type 2 diabetes were selected and 10 predictors, including systolic blood pressure, diastolic blood pressure, BMI, age, gender, duration of diabetes, family history of diabetes, whether there was a blood glucose test, whether there was exercise, and whether there was insulin treatment, were included. Then, four prediction models (SVM, DT, artificial neural network, and LR) were constructed, respectively. The model based on SVM showed the best performance with an accuracy of 79.5% and an *AUC* of 0.839. Mo et al. (5). included 4170 diabetic patients in a cross-sectional study to predict the risk of DR. Seven input variables, including age, diabetes duration, postprandial blood glucose, HbA1c, urine creatinine, urine microalbumin, and systolic blood pressure were used to construct a multivariate logistic regression model. The model showed moderate predictive ability with an *AUC* of 0.715 in the validation set. The predictive models mentioned above were developed to identify patients with DR rather than predict the occurrence of DR in the future. Therefore, they can hardly be adapted to the scenarios that require pre-diagnosis screening and prompt intervention. To our knowledge, only one follow-up study was performed for predicting the occurrence of DR (32). The study was conducted with 5034 type 2 diabetes patients not affected by retinopathy at the time of the recruitment and a median follow-up time of 1.2 years. A *Cox* risk prediction model was constructed with seven predictors including diabetes duration, HbA1c, systolic blood pressure, diastolic blood pressure, proteinuria, creatinine clearance, and diabetes drug treatment, and showed a helpful predictive ability (*C*-index=0.746). However, this model cannot meet all the clinical requirements of long-term management of type 2 diabetes and associated complications considering that it can only predict the occurrence of DR in the next 1 to 4 years.

The XGBoost model we constructed performed well in different follow-up periods (*AUC*=0.834-0.966), which indicates that it could predict whether and when a patient with type 2 diabetes will develop DR in the next 1 to 8 years or even over 8 years. Results also show the proposed model is effective to demonstrate in patients who had DR of different severity levels during follow-up. For the individuals with moderate NPDR, the model exhibited the highest performance. As a transitional stage, moderate NPDR can progress and advance to vision-threatening retinopathies. Intervention methods such as intravitreous anti-VEGF treatment can be applied to secure moderate NPDR patients. As the patient progresses to severe NPDR and PDR, it is hard to get the same treatment effect. Moreover, the method requires no extra laboratory tests since the model input was drawn from demographic and clinical characteristics and routine test results. Therefore, the model will facilitate clinicians risk-stratifying patients with type 2 diabetes. For patients at risk of DR, clinicians can provide adequate follow-up management and effective therapeutic intervention. While for the low-risk population, visit frequency will be appropriately reduced, and thus personal and societal healthcare burdens will be reduced accordingly. We further developed nomograms to depict the association between the clinical variables and the probabilities of DR. The nomogram integrating the XGBoost-based machine learning output achieved a higher predictive performance, which provides an intuitive way to interpret the model and shows its potential to be a clinical decision support tool.

To identify the key features contributing to the pre-diagnosis of DR, the importance scores of the risk factors were calculated by XGBoost. According to the feature importance analysis, HbA1c, diabetes duration, age, and FBG were highly ranked, which corresponds to the current clinical perceptions. In addition, we found that SUA, LDL-C, TC, eGFR, and TG may have the potential to be strong predictors for DR. Moreover, we further calculated the importance scores of the risk factors by RF, and found that the results were similar to and complementary to those from XGBoost. In previous studies, Krizova et al. found that an elevated level of vitreous uric acid was closely related to the development of DR, and uric acid concentration was closely related to the degree of DR progression (33). Zhu et al. found that SUA in the vitreous activates the expression of retinal inflammatory factors through the Notch signaling pathway, which will induce oxidative stress and inflammatory response and thus promote the occurrence and development of DR (34). Obesity is a metabolic disease that is associated with insulin resistance and diabetes mellitus (35). In the Wisconsin Epidemiologic Study of Diabetic Retinopathy (WESDR), it was found that high TC levels could increase the risk of retinal hard exudation in patients with type 2 diabetes (36). High LDL-C and high TC/LDL-C levels were associated with retinal macular edema in SN-DREAMS studies (37). Besides, statin lipid-lowering drugs could significantly reduce the risk of DR (38). Since DR is a microvascular complication of diabetes, eGFR can be used as an important biochemical indicator reflecting DR. Chen et al. found that higher eGFR was positively correlated with the risk of DR (39). Therefore, the results presented in our study indicated that these risk factors could be used as early and effective predictors of DR for type 2 diabetes patients before recognizable symptoms appear.

There are still several limitations to this study. First, the prediction models were constructed with the risk factors extracted from EHR, except for those with excessive missing data including the ones that might be related to the occurrence of DR (e.g., hip circumference and neck circumference). Secondly, individualized management approaches for type 2 diabetes and associated complications were not fully described. The monitoring and tracking features were also not collected during the follow-up period. The above information will be recorded and used to optimize the XGBoost model in the future. In addition, this is a single-center retrospective study. We are planning to collect cases from several institutions and conduct a prospective study to validate the generalization ability of the proposed model.

In summary, we developed and evaluated an ML-based model for predicting the risk of DR. The results suggest that it is possible to pre-diagnose DR without fundus images. We also demonstrated the potential of applying XGBoost models to facilitate clinicians in accurately identifying the high-risk population for DR and formulating patient-specific management strategies, thereby reducing the occurrence and development of DR.

## Data Availability Statement

The original contributions presented in the study are included in the article/**Supplementary Material**. Further inquiries can be directed to the corresponding authors.

## Ethics Statement

The studies involving human participants were reviewed and approved by Ethics Committee of Dalian Central Hospital. The ethics committee waived the requirement of written informed consent for participation.

## Author Contributions

XHL, ZG, and XYL conceived of and designed the study. YZ contributed to data collection and wrote the manuscript. MD contributed to the conceptualization of the project and edited the manuscript. SL contributed to discussion and revision of the manuscript. HY contributed to model building and wrote the corresponding manuscript. WC contributed to the modeling scheme design and technical support. MZ, PL, and QY contributed to part of manuscript proofreading. XHL, ZG, and XYL are the guarantor of this project and had full access to all of the data in the study and accuracy of the data analysis and reviewed the manuscript. All authors contributed to the article and approved the submitted version.

## Funding

This work was funded by the National key R&D Program of China (2016YFC0901200), the Dalian Medical Science Research Project Grant Support (2011007).

## Conflict of Interest

MD and WC are employed by Infervision.

The remaining authors declare that the research was conducted in the absence of any commercial or financial relationships that could be construed as a potential conflict of interest.

## Publisher’s Note

All claims expressed in this article are solely those of the authors and do not necessarily represent those of their affiliated organizations, or those of the publisher, the editors and the reviewers. Any product that may be evaluated in this article, or claim that may be made by its manufacturer, is not guaranteed or endorsed by the publisher.
