# Supplementary material for: Using Machine Learning Techniques to Develop Risk Prediction Models for the Risk of Incident Diabetic Retinopathy Among Patients With Type 2 Diabetes Mellitus: A Cohort Study
Source: Front Endocrinol (Lausanne). 2022 May 17;13:876559. doi: 10.3389/fendo.2022.876559 (PMC9152028; doi:10.3389/fendo.2022.876559)
Supplement: Supplementary file 1 [file DataSheet_1.docx]

Supplementary Material

# Supplementary Figures and Tables

Table S1. Candidate predictor variables.

| Attribute number | Attribute name | Attribute description | Attribute type and measurement |
| --- | --- | --- | --- |
| 1 | Age | Age of patients | Numeric(years) |
| 2 | Sex | Sex of patients | Categorical  1 = Male; 0 = Female |
| 3 | Insulin | Use Insulin or not | Categorical  1 = use Insulin;  0 = not use Insulin |
| 4 | ACEI | Use ACEI or not | Categorical  1 = use ACEI;  0 = not use ACEI |
| 5 | ARB | Use ARB or not | Categorical  1 = use ARB;  0 = not use ARB |
| 6 | Diuretic | Use Diuretic or not | Categorical  1 = use Diuretic;  0 = not use Diuretic |
| 7 | Statins | Use Statins or not | Categorical  1 = use Statins;  0 = not use Statins |
| 8 | Fibrates | Use Fibrates or not | Categorical  1 = use Fibrates;  0 = not use Fibrates |
| 9 | Hypertension | Whether patients have Hypertension | Categorical  1 = have hypertension;  0 = not have hypertension |
| 10 | CHD | Whether patients have [coronary](javascript:;) [heart](javascript:;) [disease](javascript:;) | Categorical  1 = have CHD;  0 = not have CHD |
| 11 | Smoking status | Smoking status of patients | Categorical  0 = never-smokers;  1 = current smokers;  2 = ex-smokers |
| 12 | Drinking status | Drinking status of patients | Categorical  0 = never-drinkers;  1 = current drinkers;  2 = ex-drinkers |
| 13 | Height | Height of patients | Numeric(cm) |
| 14 | Weight | Weight of patients | Numeric(kg) |
| 15 | BMI | Body mass index of patients | Numeric(kg/m^2^) |
| 16 | DBP | Diastolic blood pressure of patients at admission | Numeric(mmHg) |
| 17 | SBP | Systolic blood pressure of patients at admission | Numeric(mmHg) |
| 18 | ALT | Alanine amino transferase of patients | Numeric (U/L) |
| 19 | AST | [Aspartate](javascript:;) [amino](javascript:;) [transferase](javascript:;) of patients | Numeric (U/L) |
| 20 | γ-GT | γ-glutamyl transferase of patients | Numeric (U/L) |
| 21 | Scr | Serum creatinine level of patients | Numeric (umol/L) |
| 22 | eGFR | eGFR of patients | Numeric(ml/min) |
| 23 | SUA | Serum uric acid of patients | Numeric(umol/L) |
| 24 | TC | Total cholesterol of patients | Numeric(mmol/L) |
| 25 | TG | Triglycerides of patients | Numeric(mmol/L) |
| 26 | HDL-C | High density lipoprotein cholesterol of patients | Numeric(mmol/L) |
| 27 | LDL-C | Low density lipoprotein cholesterol of patients | Numeric(mmol/L) |
| 28 | FBG | Fasting plasma glucose of patients | Numeric(mmol/L) |
| 29 | HbA_1c_ | hemoglobin A_1c_ of patients | Numeric(%) |
| 30 | Duration | Diabetes duration of patients at admission date | Numeric(years) |
| 31 | Follow-up time | The time interval of the follow-up periods | Numeric(years) |

Table S2. Baseline information of patients in the training set and the test set.

| Attribute name | Training set (5559) | Test set (2384) | p value* |
| --- | --- | --- | --- |
| Age, mean (SD) | 63.47(11.31) | 63.72(11.12) | 0.3483 |
| Sex | | | 0.5555 |
| Male, n (%) | 3031(54.52) | 1282(53.26) |  |
| Female, n (%) | 2528(45.48) | 1102(45.78) |  |
| Insulin | | | 0.6223 |
| Use, n (%) | 227(4.08) | 91(3.78) |  |
| Not use, n (%) | 5332(95.92) | 2293(95.26) |  |
| ACEI | | | 0.5571 |
| Use, n (%) | 81(1.46) | 30(1.25) |  |
| Not use, n (%) | 5478(98.54) | 2354(97.8) |  |
| ARB | | | 0.5334 |
| Use, n (%) | 126(2.27) | 48(1.99) |  |
| Not use, n (%) | 5433(97.73) | 2336(97.05) |  |
| Diuretic | | | 0.5381 |
| Use, n (%) | 59(1.06) | 21(0.87) |  |
| Not use, n (%) | 5500(98.94) | 2363(98.17) |  |
| Statins | | | 0.5811 |
| Use, n (%) | 161(2.90) | 63(2.62) |  |
| Not use, n (%) | 5398(97.10) | 2321(96.43) |  |
| Fibrates | | | 0.2576 |
| Use, n (%) | 122(2.19) | 63(2.62) |  |
| Not use, n (%) | 5437(97.81) | 2321(96.43) |  |
| Hypertension, n (%) | | | 0.3898 |
| Have, n (%) | 400(7.20) | 158(6.56) |  |
| Not have, n (%) | 5159(92.80) | 2226(92.48) |  |
| CHD, n (%) | | | 0.2442 |
| Have, n (%) | 173(3.11) | 87(3.61) |  |
| Not have, n (%) | 5386(96.89) | 2297(95.43) |  |
| Smoking status | | | 0.4553 |
| Never-smokers, n (%) | 3780(68.00) | 1655(68.76) |  |
| Current-smokers, n (%) | 1287(23.15) | 526(21.85) |  |
| Ex-smokers, n (%) | 492(8.85) | 203(8.43) |  |
| Drinking status | | | 0.8389 |
| Never-drinkers, n (%) | 4306(77.46) | 1861(77.32) |  |
| Current-drinkers, n (%) | 1010(18.17) | 421(17.49) |  |
| Ex-drinkers, n (%) | 243(4.37) | 102(4.24) |  |
| Height, mean (SD) | 167.47(7.06) | 167.39(6.58) | 0.6244 |
| Weight, mean (SD) | 73.58(9.89) | 73.68(10.2) | 0.69 |
| BMI, mean (SD) | 26.26(4.18) | 26.28(3.63) | 0.8452 |
| DBP, mean (SD) | 84.52(12.53) | 84.86(12.44) | 0.2739 |
| SBP, mean (SD) | 143.56(22.94) | 144.78(22.45) | 0.02751 |
| ALT, mean (SD) | 30.27(37.07) | 30.03(38.64) | 0.7924 |
| AST, mean (SD) | 29.64(53.80) | 28.98(46.01) | 0.5798 |
| γ GT, mean (SD) | 48.34(89.49) | 46.71(95.17) | 0.4757 |
| Scr, mean (SD) | 76.10(73.53) | 76.68(77.14) | 0.7551 |
| eGFR, mean (SD) | 64.31(20.90) | 64.1(21.01) | 0.6782 |
| SUA, mean (SD) | 336.11(110.49) | 336.13(108.32) | 0.9941 |
| TC, mean (SD) | 4.89(1.05) | 4.91(1.08) | 0.4067 |
| TG, mean (SD) | 2.09(1.62) | 2.04(1.54) | 0.2033 |
| HDL-C, mean (SD) | 1.03(0.24) | 1.05(0.24) | 0.05972 |
| LDL-C, mean (SD) | 2.89(0.82) | 2.91(0.87) | 0.3677 |
| FBG, mean (SD) | 8.94(3.35) | 8.81(3.11) | 0.0845 |
| HbA_1c_, mean (SD) | 8.53(7.72) | 8.39(6.45) | 0.4057 |
| Duration, mean (SD) | 1.43(2.75) | 1.5(2.77) | 0.2822 |
| Follow up time, mean (SD) | 3.16(2.25) | 3.09(2.22) | 0.1903 |

*P value is obtained from a t-test for continuous variable and a Chi-square test for a categorical variable.

Table S3. Key hyper-parameters of the ML models.

| Model | Hyper-parameter | value |
| --- | --- | --- |
| RF | max_depth | 11 |
|  | max_features | 15 |
|  | n_estimators | 100 |
|  |  |  |
| XGBoost | n_estimators | 200 |
|  | max_depth | 5 |
|  | learning rate | 0.1 |
|  | subsample | 1 |
|  | colsample_bytree | 0.5 |
|  |  |  |
| LR | C（[penalty](javascript:;) [coefficient](javascript:;)） | 1 |
|  |  |  |
| SVM | C（[penalty](javascript:;) [coefficient](javascript:;) ） | 100 |
|  | gamma | 10 |
|  |  |  |
| K-NN | n_neighbors | 54 |

Table S4. Baseline information of patients at the index date.

| Attribute name | Overall (7943) | DR group (1692) | Non-DR group (6251) | p value* |
| --- | --- | --- | --- | --- |
| Age, mean (SD) | 63.54(11.25) | 59.60(11.04) | 64.61(11.07) | < 2.2e-16 |
| Sex | | | | 0.573 |
| Male, n (%) | 4313(54.30) | 908(53.66) | 3405(54.47) |  |
| Female, n (%) | 3630(45.70) | 784(46.34) | 2846(45.53) |  |
| Duration at index date, mean (SD) | 1.449(2.756) | 3.084(3.386) | 1.007(2.373) | < 2.2e-16 |
| Follow up time, mean (SD) | 3.139(2.243) | 4.316(2.325) | 2.820(2.111) | < 2.2e-16 |

*P value is obtained from a t-test for continuous variable and a Chi-square test for a categorical variable.

Figure S1. The importance scores of the features calculated by Random Forest.


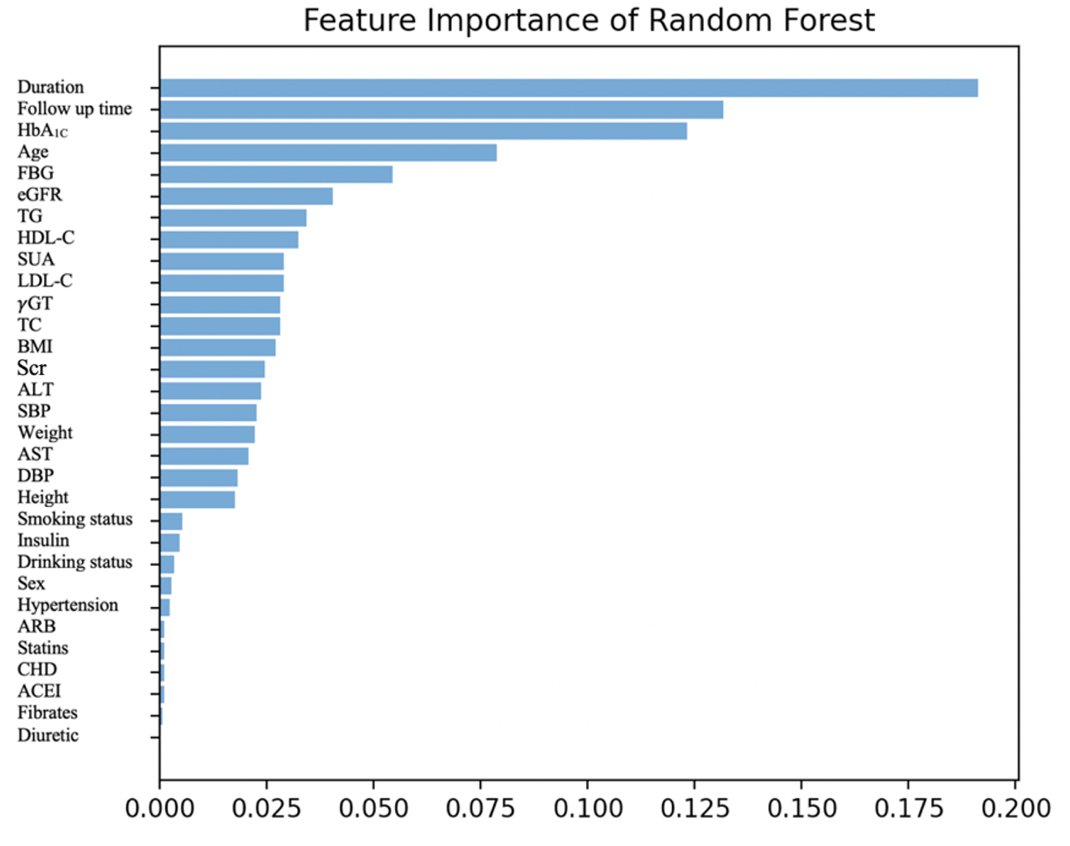


Mean Decrease Gini

**
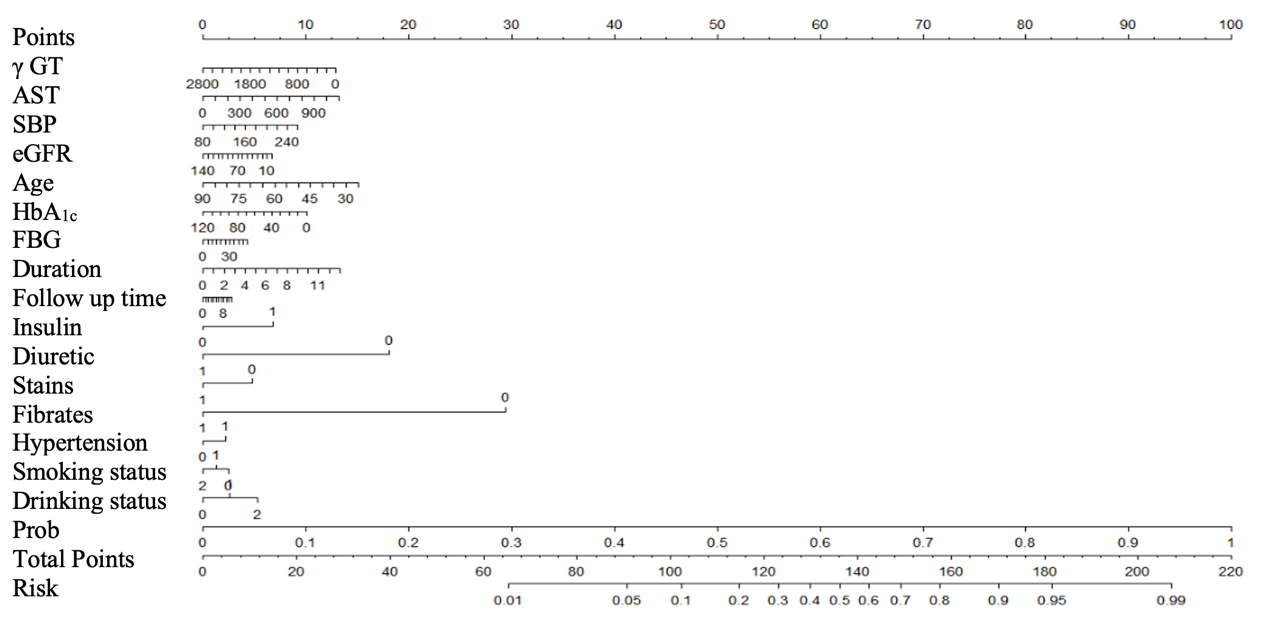
**

Figure S2. Nomogram of the LR model with the output of the XGBoost model for predicting DR risk of patients with T2DM.

**
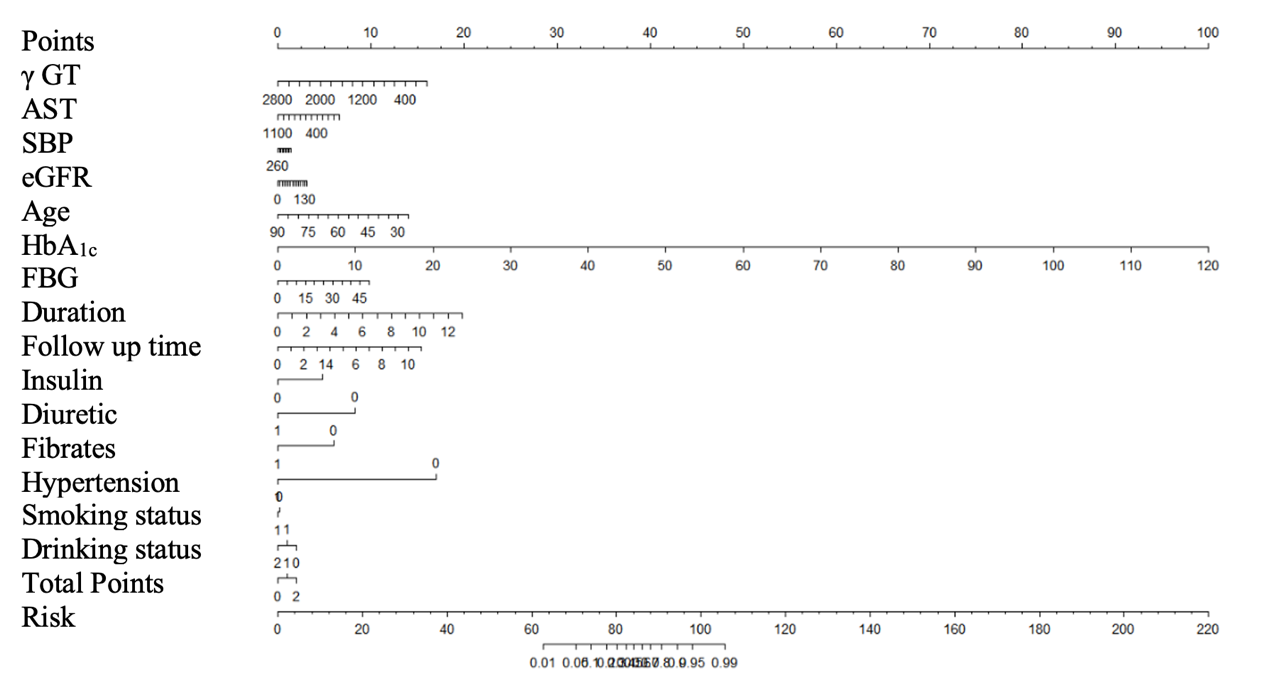
**

Figure S3. Nomogram of the classic LR model for predicting DR risk of patients with T2DM.

**Table S5. Baseline information of cases with correct and incorrect predictions in the supplementary test set.**

| **Attribute name** | **Correct set (768)** | **Incorrect set (61)** | **P value^*^** |
| --- | --- | --- | --- |
| Age, mean (SD) | 58.97(10.44) | 66.55(10.39) | 6.209e-07 |
| Sex | | | 0.7604 |
| Male, n (%) | 431(56.12) | 36(59.02) |  |
| Female, n (%) | 337(43.88) | 25(40.98) |  |
| Insulin | | | 0.5467 |
| Use, n (%) | 61(7.94) | 3(4.92) |  |
| Not use, n (%) | 707(92.06) | 58(95.08) |  |
| ACEI | | | 0.5362 |
| Use, n (%) | 2(0.26) | 1(1.64) |  |
| Not use, n (%) | 766(99.74) | 60(98.36) |  |
| ARB | | | 1 |
| Use, n (%) | 15(1.95) | 1(1.64) |  |
| Not use, n (%) | 753(98.05) | 60(98.36) |  |
| Diuretic | | | < 2.2e-16 |
| Use, n (%) | 1(0.13) | 0(0.00) |  |
| Not use, n (%) | 767(99.87) | 61(100.00) |  |
| Statins | | | 0.0002492 |
| Use, n (%) | 2(0.26) | 3(4.92) |  |
| Not use, n (%) | 766(99.74) | 58(95.08) |  |
| Fibrates | | | < 2.2e-16 |
| Use, n (%) | 1(0.13) | 0(0.00) |  |
| Not use, n (%) | 767(99.87) | 61(100.00) |  |
| Hypertension | | | 0.1276 |
| Have, n (%) | 35(4.56) | 6(9.84) |  |
| Not have, n (%) | 733(95.44) | 55(90.16) |  |
| CHD | | | 1 |
| Have, n (%) | 17(2.21) | 1(1.64) |  |
| Not have, n (%) | 751(97.79) | 60(98.36) |  |
| Smoking status | | | 0.00367 |
| Never-smokers, n (%) | 531(69.79) | 44(72.13) |  |
| Current-smokers, n (%) | 198(25.78) | 9(14.75) |  |
| Ex-smokers, n (%) | 34(4.43) | 8(13.11) |  |
| Drinking status | | | 0.7486 |
| Never-drinkers, n (%) | 588(76.56) | 46(75.41) |  |
| Current-drinkers, n (%) | 156(20.31) | 12(19.67) |  |
| Ex-drinkers, n (%) | 24(3.12) | 3(4.92) |  |
| Height, mean (SD) | 167.88(6.1) | 167.63(6.72) | 0.7826 |
| Weight, mean (SD) | 74.55(9.58) | 71.13(8.11) | 0.002505 |
| BMI, mean (SD) | 26.42(2.89) | 25.32(2.57) | 0.002166 |
| DBP, mean (SD) | 84.44(11.38) | 86.66(16.26) | 0.3007 |
| SBP, mean (SD) | 142.49(21.9) | 151(26.74) | 0.01817 |
| ALT, mean (SD) | 28.86(33.1) | 25.96(23.3) | 0.3701 |
| AST, mean (SD) | 25.34(30.86) | 26.17(21.22) | 0.78 |
| γ GT, mean (SD) | 42.39(68.56) | 33.44(21.5) | 0.01655 |
| Scr, mean (SD) | 71.3(69.4) | 90.05(103.38) | 0.1689 |
| eGFR, mean (SD) | 70.93(21.28) | 56.1(18.54) | 8.693e-08 |
| SUA, mean (SD) | 325.28(87.76) | 371.77(104.96) | 0.001263 |
| TC, mean (SD) | 5.05(1.09) | 4.88(0.98) | 0.1906 |
| TG, mean (SD) | 2.33(2.1) | 1.99(1.24) | 0.05909 |
| HDL-C, mean (SD) | 1.07(0.25) | 1.02(0.21) | 0.0672 |
| LDL-C, mean (SD) | 2.89(0.82) | 2.83(0.9) | 0.6166 |
| FBG, mean (SD) | 9.42(2.9) | 8.9(2.77) | 0.1624 |
| HbA_1c_, mean (SD) | 10.16(11.55) | 7.53(1.38) | 9.58e-09 |
| Duration, mean (SD) | 4.05(2.41) | 1.67(2.41) | 6.728e-07 |
| Follow up time, mean (SD) | 3.53(3.44) | 2.69(1.83) | 2.953e-07 |
| DR grading | | | 1 |
| 1-3, n (%) | 735(95.70) | 58(95.08) |  |
| 4-5, n (%) | 33(4.30) | 3(4.92) |  |

*P value is obtained from a t-test for continuous variable and a Chi-square test for a categorical variable.


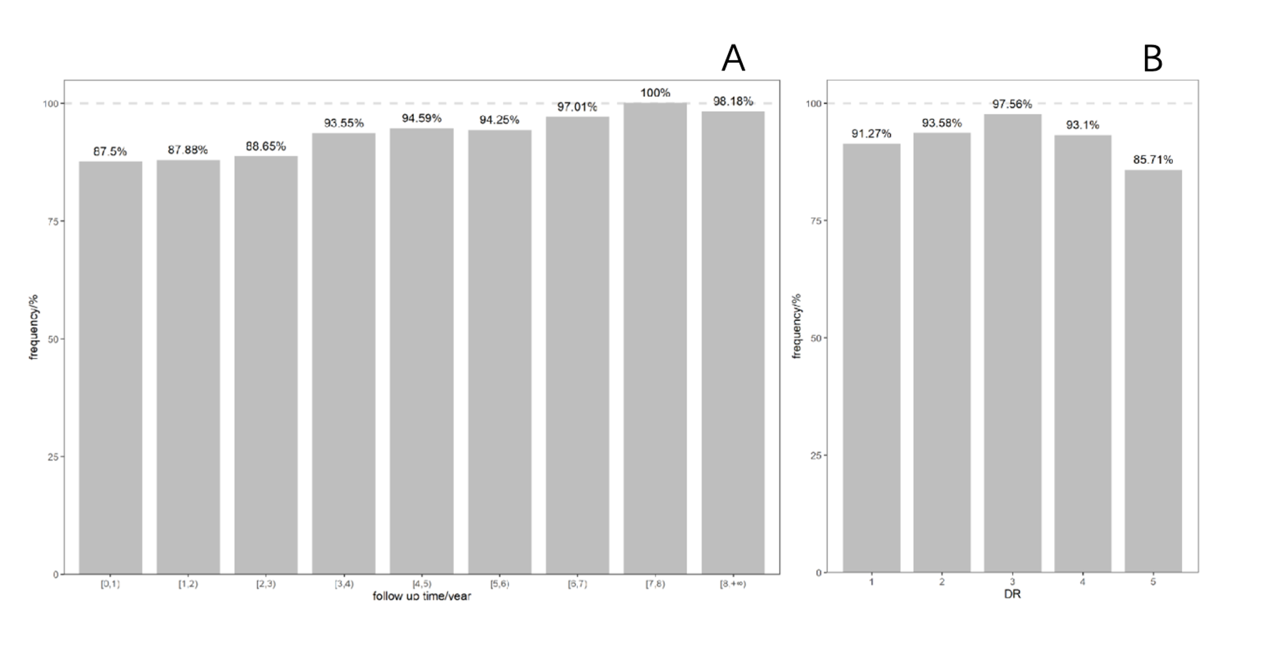


Figure S4. Proportion of cases with correct predictions for different follow-up periods (A) and for DR severity levels (B) on the supplementary data set.
